# Cellular and Molecular Targets of Menthol Actions

**DOI:** 10.3389/fphar.2017.00472

**Published:** 2017-07-18

**Authors:** Murat Oz, Eslam G. El Nebrisi, Keun-Hang S. Yang, Frank C. Howarth, Lina T. Al Kury

**Affiliations:** ^1^Department of Pharmacology, College of Medicine and Health Sciences, United Arab Emirates University Al Ain, United Arab Emirates; ^2^Department of Basic Medical Sciences, College of Medicine, Qatar University Doha, Qatar; ^3^Department of Biological Sciences, Schmid College of Science and Technology, Chapman University Orange, CA, United States; ^4^Department of Physiology, College of Medicine and Health Sciences, United Arab Emirates University Al Ain, United Arab Emirates; ^5^Department of Health Sciences, College of Natural and Health Sciences, Zayed University Abu Dhabi, United Arab Emirates

**Keywords:** monoterpenes, voltage-gated ion channels, ligand-gated ion channels, TRP channels, menthol

## Abstract

Menthol belongs to monoterpene class of a structurally diverse group of phytochemicals found in plant-derived essential oils. Menthol is widely used in pharmaceuticals, confectionary, oral hygiene products, pesticides, cosmetics, and as a flavoring agent. In addition, menthol is known to have antioxidant, anti-inflammatory, and analgesic effects. Recently, there has been renewed awareness in comprehending the biological and pharmacological effects of menthol. TRP channels have been demonstrated to mediate the cooling actions of menthol. There has been new evidence demonstrating that menthol can significantly influence the functional characteristics of a number of different kinds of ligand and voltage-gated ion channels, indicating that at least some of the biological and pharmacological effects of menthol can be mediated by alterations in cellular excitability. In this article, we examine the results of earlier studies on the actions of menthol with voltage and ligand-gated ion channels.

## Introduction

Menthol, a naturally occurring cyclic monoterpene alcohol of plant origin, gives plants of the Mentha species their distinctive smell and flavor. In Japan for more than 2,000 years, peppermint plant, the main source of menthol, has been cultivated for medicinal purposes (Patel et al., 2007). Menthol is also an important constituent of essential oils such as eucalyptus, lemongrass, and palmarosa. In the present day, menthol is widely used in oral hygiene products, confectionary, pharmaceuticals, cosmetics, pesticides, and as a flavoring agent. With regards to its medicinal purposes, both prescribed and over-the-counter menthol containing medications are currently available for a host of conditions, including respiratory diseases, gastrointestinal disorders, common cold, and musculoskeletal pain (Eccles, 1994; Patel et al., 2007). In addition, it is commonly used as part of analgesic, antiseptic, topical antipruritic, and cooling formulations. It is estimated that ~30,000 metric tons of menthol are consumed annually (Kamatou et al., 2013). After citrus and vanilla, menthol is one of the most important flavoring substances in culinary industry. It is also a commonly used compound in many tobacco products. The estimated use pattern for L-menthol at the beginning of twenty-first century is as follows: 36% oral care products, 22% pharmacy products, 19% tobacco products, 17% flavors, 6% others (OECD, 2003). Despite menthol's use since antiquity, the mechanisms mediating its pharmacological actions remain relatively unknown. The focus of this report is to highlight the recent advances in the menthol research and to provide an overview of its actions on cellular excitability.

**Figure 1:**
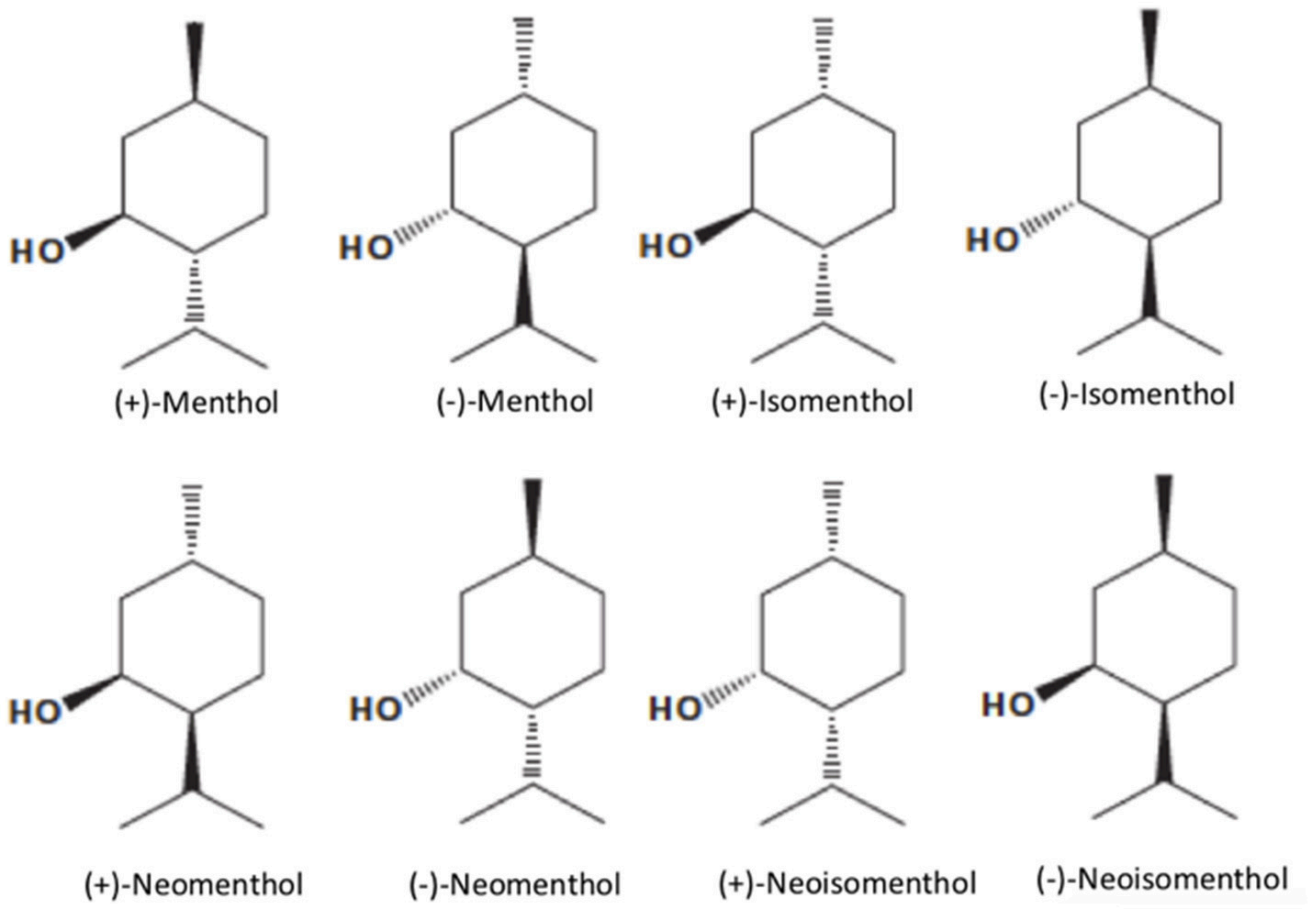
Structure of menthol isomers (from left to right, top row): (+)-Menthol, (−)-Menthol, (+)-Isomenthol, (−)-Isomenthol, (+)-Neomenthol, (−)-Neomenthol, (+)-Neoisomenthol, (−)-Neoisomenthol.

Menthol [5-methyl-2-(1-methylethyl) cyclohexanol; 2-isopropyl-5-methylcyclohexanol or p-methan-3-ol], with the molecular formula C10H20O (MW. 156.27), is a natural compound with three asymmetric carbon atoms and, thus, occurs as four pairs of optical isomers namely, (+)- and (−)-menthol, (+)- and (−)-neomenthol, (+)- and (−)-neoisomenthol, and (+)- and (−)-isomenthol (Figure 1). The major form of menthol found in nature is (−)-menthol (L-menthol). (−)-Menthol is frequently employed since it retains better cooling properties than the other isomers (Figure 1). Menthol is a colorless or white, crystalline, and flaky substance. Depending on its purity, menthol has a melting point of 41–44°C and it is solid at room temperature (25°C) with a density of 0.890 g/cm^3^. It is not completely soluble in water (431 mg/L at 20°C), but freely soluble in chloroform, diethyl ether, and alcohol (Kamatou et al., 2013) with Log_octanol/water_ value of 3.4, indicating that menthol is a lipophilic compound capable of interacting with membranes (Turina et al., 2006).

All menthol isomers are absorbed well through oral route of exposure and are excreted mainly as glucuronides. In rats, an extensive enterohepatic circulation additionally leads to various hydroxylated degradation products. Main elimination pathway for glucuronides and degradation products is via urine; however, small quantities are removed via feces. For all isomers of menthol, a very low acute oral toxicity with LD_50_ values normally greater than 2,000 mg/kg bw has been reported. In an earlier report, rats receiving diets with up to 200 mg/kg bw/d of menthol for 5.5 weeks showed no signs of toxicity (National Toxicology Program, 1979; OECD, 2003).

## Effects of menthol on ion channels

Ion channels are pore-forming integral membrane proteins that regulate the transfer of charged ions (Ca^2+^, K^+^, Na^+^, or Cl^−^) through the bilayer membrane of the cell and regulate resting membrane potential, action potentials and other electrical signals. Channel opening is usually triggered by a specific stimulus such as membrane depolarization, mechanical stretch, or binding to a ligand. Ionic currents produced by the passage of ions through these channels constitutes the electrophysiological origin for several cellular events including the release of neurotransmitters, muscle contraction, cell development, secretion of hormones, various transmembrane signaling processes, and excitation-transcription coupling (Cannon et al., 2014; Tien et al., 2014). It is known that several chemicals demonstrate their therapeutic actions by influencing the functions of ion channels in different types of cells. In recent years, there has been considerable interest in pharmacological targets of menthol. Actions of menthol on various ion channels have been reported in several recent investigations. The results of some of these recent studies will be reviewed in the following sections (Table 1).

**Table 1:** Summary of cellular and molecular effects of menthol.

**Target protein or cellular event**	**End-point measured**	**Effect**	**Concentration and ligand**	**Preparation**	**References**
Na^+^ channels	Ion current	Inhibition	IC_50_ = 376 μM (In neuronal Na^+^ channels)	HEK293 cells	Haeseler et al., 2002
			IC_50_ = 571 μM (In skeletal muscle Na^+^ channels)		
Na^+^ channels	Ion current	Inhibition	IC_50_ = 297 μM	Cultured dorsal horn neurons	Pan et al., 2012
TTX-resistant Na^+^ channels	Ion current	Inhibition	IC_50_ = 299–500 μM	DRG neurons and immortalized DRG neuron-derived F11 cells	Gaudioso et al., 2012
Nav1.8 channel subtype			>300 μM		
			540–807 μM		
Nav1.9 channel subtype					
TTX-sensitive Na^+^ channels					
Na^+^ channels	Compound action potential	Inhibition	IC_50_ = 1.1 mM (−)-menthol	Frog sciatic nerve fibers	Kawasaki et al., 2013
			IC_50_ = 0.9 mM (+)-Menthol		
Na^+^ channels	Number and duration of action potential bursts	Inhibition	250 μM	Mouse cortical neurons	Pezzoli et al., 2014
DHP-sensitive and DHP-insensitive Ca^2+^ channels	Ion current	Inhibition	IC_50_ = 0.25 mM	LA-N-5 cells	Sidell et al., 1990
Ca^2+^ channels	High K^+^-induced intracellular Ca^2+^ increase	Inhibition	2 mM	Leech neurons	Dierkes et al., 1997
Ca^2+^ channels	Ca^2+^ uptake and contractile response	Inhibition	IC_50_ = 8–28 μg/ml	Ilium	Hawthorn et al., 1988
			IC_50_ = 10–69 μg/ml	Cardiac tissue synaptosomes and chick retinal neurons	
Ca^2+^ channels	KCl-preconstricted smooth muscle contraction	Inhibition	IC_50_ = 58 μM	Bronchial smooth muscle fibers	Wright et al., 1997
			IC_50_ = 120 μM		
	ACh-preconstricted smooth muscle contraction				
Ca^2+^ channels	Intracellular Ca^2+^	Inhibition	0.01–1 mM	Tracheal smooth muscle fibers	Ito et al., 2008; Wang et al., 2016
Ca^2+^ channels	Intracellular Ca^2+^	Inhibition	300 μM	Detrusor muscle	Ramos-Filho et al., 2014
Ca^2+^ channels	Intracellular Ca^2+^	Inhibition	0.1–1 mM	Vas deference	Filippov et al., 2009; Vladymyrova et al., 2011
Ca^2+^ channels	Ca^2+^ influx and smooth muscle relaxation	Inhibition	0.1–1 mM	Rat aorta, mesenteric and coronary arteries	Cheang et al., 2013
Ca^2+^ channels	Ca^2+^ influx and contraction	Inhibition	0.1–30 mM	Gastrointestinal smooth muscle and human colon circular muscle	Amato et al., 2014a
Ca^2+^ channels	High K^+^ and Ca^2+^ evoked contractions	Inhibition	IC_50_ = 22.1 μg/mL	Guinea pig taenia coli	Hills and Aaronson, 1991
			IC_50_ = 25.9 μg /mL	Guinea pig colon	
			IC_50_ = 15.2 μg /mL	Rabbit jejunum	
Ca^2+^ channel	Ca^2+^ current	Inhibition	0.1–0.5 mM	Helix neurons	Swandulla et al., 1986
Low voltage-activated Ca^2+^ channel (T-type like) and high voltage activated Ca^2+^ channel (L-type like)	Ca^2+^ current	Inhibition	0.1–1 mM	Cultured DRG neurons	Swandulla et al., 1987
L-type VGCCs	Ca^2+^ current	Inhibition	IC_50_ = 74.6	Rabbit ventricular myocyte	Baylie et al., 2010
TRPM8	Ca^2+^ current	Activation	300 μM	Rat tail artery myocytes	Melanaphy et al., 2016
L-type VGCCs		Inhibition			
IP3 receptors		Activation			
Ryanodine receptors	Ca^2+^ efflux	Activation	EC_50_ =1 mM	Isolated sarcoplasmic reticulum	Palade, 1987; Mahieu et al., 2007; Neumann and Copello, 2011
				HEK239 cells	
Ca^2+^-activated K^+^ channels	Ion current	Activation	100 μM	Human glioblastoma cells	Wondergem and Bartley, 2009
Kv7.2/3 channel	Ion current	Suppression	IC_50_ = 289 μM	Cultured sensory neurons	Vetter et al., 2013
TRPM8 channels	Intracellular Ca^2+^	Activation	10 μM and 100 μM	CHO cells	McKemy et al., 2002; Peier et al., 2002
			EC_50_ = 66.7 μM	*Xenopus laevis* oocytes	
TRPM8 channels	Menthol sensitivity of TRPM8 channel	Altered	10, 100, and 1000 μM	Mutant voltage sensor residues (TM4 and TM4-TM5 linker) of human TRPM8 channel expressed in HEK 293 cells	Voets et al., 2007
TRPM8 channels	Menthol sensitivity to TRPM8 channel	Reduction	300 μM	Mutant tyrosine 745 residue in TM2 of mouse TRPM8	Bandell et al., 2006
TRPM8 channels	Ion current	Activation	EC_50_ = 4–80 μM	Trigeminal ganglia neuronal cells	McKemy et al., 2002; Peier et al., 2002; Behrendt et al., 2004
				CHO Cells	
				HEK293 calls	
TRPV3 channels	Ion current	Activation	0.5–2 mM	CHO cells	Macpherson et al., 2006; Vogt-Eisele et al., 2007
			1 mM	HEK293 cells	
				Primary keratinocyte culture	
TRPA1 channels	Ion current	Activation (at low concentration)	1–30 μM	CHO cells	Macpherson et al., 2006; Karashima et al., 2007; Kim et al., 2016
			1–10 μM	Interstitial cells of Cajal	
				CHO cells	
		Inhibition (at high concentration)	0.25–1 mM		
TRP-independent effects	Intracellular Ca^2+^	Inhibition	EC50 = 0.9–1 mM	Skeletal muscle sarcoplasmic reticulum	Palade, 1987; Takeuchi et al., 1994; Tsuzuki et al., 2004; Lu et al., 2006; Mahieu et al., 2007; Wondergem and Bartley, 2009; Neumann and Copello, 2011
			100 μM–1 mM	Tracheal epithelial cells	
			25–100 μM	Human leukemia cells	
			100 μM	Gliablastoma cells	
			100 μM–1 mM	Cell lines and dorsal horn neurons	
PCL enzyme activity	P2Y purinoreceptor-mediated/histamine receptor-mediated cytosolic Ca^2+^ mobilization	Inhibition	0.3–1 mM	HEK-293 cells	Kim et al., 2008
				HeLa cells	
Anion transport	CFTR-mediated Cl^−^ transport	Potentiation	0.1–1 mM	Human airway Calu-3 epithelial cells	Morise et al., 2010
	Na^+^-K^+^-2Cl^−^ transporter	Down regulation			
GABA_A_ (α1β2γ2s) receptors	Ion current	Potentiation	EC_50_ = 25 μM	*Xenopus laevis* oocytes	Hall et al., 2004
Glycine (α1 homomers) receptors			EC_50_ = 75 μM		
GABA_A_ receptors	Righting reflex	Inhibition	EC_50_ = 23 μM	*In vivo* tadpole assay	Watt et al., 2008
GABA_A_ receptors	[^3^H]-flunitrazepam binding	No effect	Up to 500 μM	Cultured mouse cortical neurons	García et al., 2006; Corvalán et al., 2009
		Stimulation	EC_50_ = 1.55 μM	Membranes from chick forebrain	
GABA_A_ receptors	[^3^H]-TBOB binding	Inhibition	LD_50_= 128.9 μg fly^−1^	Housefly head membrane preparations	Tong and Coats, 2012
GABA_A_ receptors	Ion current	Activation	100 μM	Cultured rat hippocampal neurons	Zhang et al., 2008
GABA_A_ receptors	Ion current	Activation	0.1–1 mM	brainstem-spinal cord of newborn rats	Tani et al., 2010
GABA_A_ receptors	Ion current	Activation	150–750 μM	Periaqueductal gray (PAG) neurons of midbrain slices	Lau et al., 2014
5-HT_3_ receptors	[^14^C] guanidinium influx	Inhibition	10 μM–1 mM	N1E-115 cells	Heimes et al., 2011
	Isotonic contractions	Inhibition		Isolated rat ileum	
5-HT_3A_ receptors	Ion current	inhibition	IC_50_ = 163 μM.	*Xenopus laevis* oocytes	Ashoor et al., 2013a
Human recombinant homomeric 5-HT_3A_	Ca^2+^ influx	Inhibition	IC_50_ = 4.75 mM for (−)-menthol	HEK293 cells	Walstab et al., 2014
Human recombinant heteromeric 5-HT_3AB_ receptors			IC_50_ = 4.75 mM for (+)-Menthol		
			IC_50_ = 4.46 mM for (−)-menthol		
			IC_50_ = 4.60 mM for (+)-Menthol		
Nicotinic receptors	Nicotine-induced irritation and sensory perception	Reduction	0.3% L-menthol	Tongue (human subject)	Dessirier et al., 2001
Nicotinic receptors	Respiratory irritation response	Reduction	16 ppm	Female C57BL/6J mice	Willis et al., 2011
Nicotinic receptors	nicotine-induced hypothermia	Inhibition	100-400 mg/Kg	Male adult rats	Ruskin et al., 2008
Nicotinic receptors	Gastric relaxation	Induction	0.3-30 mM	Male adult mice	Amato et al., 2014b
Nicotinic receptors (α4β2 nAChRs)	Ion current	Inhibition	IC_50_ = 111 μM.	Trigeminal neurons in HEK tsA201 cells	Hans et al., 2012
Nicotinic receptors (α4β2 nAChRs)	Density of α4β2 nAChR in menthol smokers	Up-regulation	Menthol cigarette smokers vs nonsmokers	Human subjects	Brody et al., 2013
Nicotinic receptors subunits	α4 and α6 nAChR subunits	Up-regulation	2 mg/kg/h for *in vivo* upregulation assays	Midbrain dopaminergic neurons from mice	Henderson et al., 2016
	(α4)3(β2)2 nAChR subunits	Up-regulation	500 nM	Neublastoma cells	
	Decay phase of current	Acceleration	2 mg/kg/10d	Mouse brain slices	
Nicotinic receptors (α7 nAChRs)	Ion current	Inhibition	IC_50_ = 32.6 μM	*Xenopus laevis* oocytes	Ashoor et al., 2013b
Nicotinic receptors (α3β4 nAChRs)	Ion current	Desensitization	Up to 1 mM	HEK 293 cells and mouse sensory neurons	Ton et al., 2015

### Voltage-gated sodium channels

Voltage-gated sodium channels (VGSCs) have essential roles in the generation and propagation of action potentials (APs) in excitable cells including neurons, cardiomyocytes, smooth muscle cells, and skeletal muscle fibers (Savio-Galimberti et al., 2012; Peters and Ruben, 2014). VGSCs are stimulated by the changes in membrane potential. The depolarizing changes are detected by a voltage sensor connected to the pore domain that opens and allows the influx of sodium. Local anesthetics, antinociceptive, antiarrhythmic, and antiepileptic drugs antagonize the functions of ion channels by binding to a specific site located on the protein structure, whereas other classes of chemicals and neurotoxins bind to distinct receptor sites within the pore of the channel (for a review, Catterall et al., 2013).

Various over-the-counter products for pain relief and topical balms often contain menthol in the concentrations ranging from 5 to 16% (320–1,024 mM). In this concentration range, topical application of menthol has been shown to be antinociceptive for heat and cold-induced pain (Green, 1986; Albin et al., 2008; Klein et al., 2010, 2012). Menthol has also been shown to have anti-nociceptive actions in the mouse abdominal constriction and hot-plate tests (Galeotti et al., 2002). Anti-nociceptive actions of menthol were stereo-selective, since only (−) enantiomer was active in *in vivo* models. Local anesthetic activity of menthol has also been demonstrated in earlier studies (Galeotti et al., 2001). However, both (−) and (+) isomers of menthol are equally active in their local anesthetic actions.

The effect of menthol on sodium channels was tested in an earlier patch-clamp study (Haeseler et al., 2002) in HEK293 cells transfected with human skeletal muscle and rat neuronal VGSCs. Menthol suppressed whole cell Na^+^ currents with an IC_50_ value of 571 and 376 μM for neuronal cells and skeletal muscle fibers, respectively. The strength of menthol effects enhanced significantly at depolarized potentials with increasing portion of inactivated channels, suggesting that preferential blockade of VGSC in inactivated state may mediate antinociceptive and local anesthetic effects of menthol (Haeseler et al., 2002). In another electrophysiological study, Gaudioso et al. (2012), using whole-cell patch clamp method, investigated the actions of menthol on tetrodotoxin (TTX)-sensitive VGSCs in immortalized dorsal root ganglia (DRG) neuron-derived F11 cells and on TTX-resistant Nav1.9 and Nav1.8 channel subtypes in DRG neurons, and (Gaudioso et al., 2012). It was found that VGSCs were suppressed by menthol in a concentration, frequency, and voltage-dependent manner. Firing at high-frequency stimulation was suppressed by menthol while minimal effect was observed on normal neuronal activity recorded by current clamp technique. In addition, menthol at low concentrations caused analgesia and relieved pain produced by a Na^+^ channel-targeting toxin in mice. In conclusion, the results of this study indicated that Nav1.8, Nav1.9, and TTX-sensitive Na^+^ channels are blocked by menthol in a state-selective manner suggesting a role for Na^+^ channel blockade in the efficacy of menthol as topical analgesic compound (Gaudioso et al., 2012).

Menthol, by dose-dependently diminishing both contralateral and ipsilateral pain hypersensitivity produced by complete Freund's adjuvant, showed direct effects on the spinal cord (Pan et al., 2012). In addition, both first and second stages of formalin-induced spontaneous nocifensive behavior were attenuated by menthol. In cultured dorsal horn neurons, menthol inhibited VGSCs in a state-, use-, and voltage-dependent manner. Moreover, menthol inhibited repetitive firing and the amplitudes of action potentials, inhibited spontaneous synaptic transmission and decreased excitability in cultured superficial dorsal horn neurons. The examination of brain menthol concentrations showed that, when applied systemically, menthol is rapidly accumulated in the brain tissue (Pan et al., 2012), suggesting that this compound induces analgesic effect on inflammatory pain models via inhibition of VGSCs in the central nervous system. In another study (Kawasaki et al., 2013), both (−) and (+)-menthol concentration-dependently decreased the peak amplitudes of compound action potentials (CAPs) with the IC_50_ values of 1.1 and 0.93 mM, respectively. (−)-menthone and (+)-menthone also suppressed CAPs with extents similar to that of menthol (Kawasaki et al., 2013).

A recent study in mouse cortical neurons (Pezzoli et al., 2014) reported that menthol (250 μM) dampens the generation of action potentials in a time- and voltage-dependent manner in TRPM8 knock-out mice and in the presence of a TRPM8 blocker. The effects of menthol were also studied on seizures induced by *in vitro* gabazine applications. Menthol decreased the duration and the number of action potential bursts. In addition, an increase in the concentration of gabazine was needed to elicit seizures. The results suggest that menthol can modulate VGSCs of cortical neurons in the brain through a TRP-independent pathway (Pezzoli et al., 2014).

### Voltage-gated calcium channels

Voltage-gated Ca^2+^ channels (VGCCs) are required for key functions in excitable cell. (Hofmann et al., 2014; Simms and Zamponi, 2014). The opening of VGCCs by changes in membrane depolarization causes rapid increases in cytoplasmic Ca^2+^ concentration. Elevated Ca^2+^ levels can trigger key cellular events such as contraction, exocytosis, gene transcription, and excitability (Badou et al., 2013; Bannister and Beam, 2013; Reuter et al., 2013).

Several earlier reports have demonstrated that menthol modulates the functional properties of VGCCs. In LA-N-5 cells, brief application of menthol inhibited the depolarization-induced Ca^2+^ influx though both dihydropyridine-sensitive and -insensitive L-type Ca^2+^ channels with IC_50_ value of 0.25 mM (Sidell et al., 1990). Ca^2+^ increases induced by high K^+^ were suppressed by menthol in Leech neurons (Dierkes et al., 1997), chick retinal neurons and synaptosomes (Hawthorn et al., 1988). It was shown that menthol suppresses high K^+^-elicited and electrically stimulated contractile responses in atrial and papillary muscles and in ileum. IC_50_ values in the ileum tissue ranged from 8 to 28 μg/ml and in the cardiac preparations from 10 to 69 μg/ml. Notably, menthol acts as a competitive inhibitor on the specific binding of [^3^H]PN 200-110 and [^3^H] nitrendipine, dihydropyridine class antagonists of L-type Ca^2+^ channels in cardiac and smooth muscles and neuronal tissue with potencies similar to those determined in earlier studies (Hawthorn et al., 1988). In tracheal (Ito et al., 2008) and bronchial (Wright et al., 1997) smooth muscle fibers, vas deference (Filippov et al., 2009; Vladymyrova et al., 2011), and detrusor muscle (Ramos-Filho et al., 2014), menthol decreased the KCl- and ACh-induced muscle contractures by inhibiting of L-type VGCCs. Recent study in tracheal smooth muscle preparation reported that methacholine and electrical field stimulation induced contractions were suppressed significantly by menthol and suggested that VGCCs were affected by this compound (Wang et al., 2016).

Consistent with earlier findings, menthol was demonstrated to cause relaxation and suppress contraction in coronary and mesenteric arteries, and rat aorta (Cheang et al., 2013), mainly by decreasing the influx of Ca^2+^ via dihydropyridine-sensitive L-type VGCCs. In addition, menthol (0.1–30 mM) inhibited the contractility of the gastrointestinal smooth muscle and induced spasmolytic effects in human colon circular muscle by inhibiting the entrance of Ca^2+^ through L-type VGCCs (Amato et al., 2014a). Peppermint oil has also been shown to reduce the contractions evoked by high K^+^ and Ca^2+^ in guinea pig taenia coli by inhibiting L-type VGCCs (Hills and Aaronson, 1991).

Several electrophysiological studies investigated the effects of menthol on currents mediated by VGCCs. In a previous investigation, the effects of menthol (0.1–0.5 mM) on the inactivation of Ca^2+^ currents were examined in Helix neurons (Swandulla et al., 1986). Although internal application was ineffective, external application of menthol caused acceleration of inactivation during Ca^2+^-dependent rapid phase. In following experiments, the actions of menthol on Ca^2+^ current were examined in cultured DRG neurons from embryos of rat and chick (Swandulla et al., 1987). Bath application of menthol (0.1–1 mM) caused various types of effects on different kinds of Ca^2+^ currents in these neurons. Below membrane potential of −20 mV, menthol suppressed the amplitudes of low threshold (T-type like) Ca^2+^ currents in a dose-dependent fashion; with no alteration of activation kinetics. On the other hand, externally applied menthol significantly accelerated the inactivation of the L-type (high-threshold) Ca^2+^ currents (activated from a holding potential of −80 mV to positive potentials above −20 mV). The effects of menthol remained unaltered at holding potentials more positive than −20 mV. Importantly, the effect of menthol was observed only when it was applied from the outside. Together, findings of this investigation showed that menthol inhibits Ca^2+^ influx through the low voltage-activated Ca^2+^ channel, and enhances the inactivation of the L-type (high voltage-activated) Ca^2+^ channel.

Succeeding these investigations in neurons, the effects of menthol on L-type VGCCs of rabbit ventricular myocyte was studied using whole-cell recording technique at near-physiological temperature (~35°C) (Baylie et al., 2010). Menthol inhibited peak amplitudes of Ca^2+^ currents in a concentration-dependent manner with an IC_50_ value of 74.6 μM. The late currents remaining at the end of depolarizing pulses were also suppressed by menthol. Menthol inhibited these late currents with greater efficacy (96% block at 1 mM) than peak (68% block at 1 mM) Ca^2+^ currents (Baylie et al., 2010). Similarly, inhibition of L-type VGCCs by menthol has also been shown in smooth muscle cells (Melanaphy et al., 2016).

The effects of menthol are not limited to Ca^2+^ influx through VGCCs. Menthol has significant effect on other components of Ca^2+^ homeostasis as well. For example, it has been shown that (Mahieu et al., 2007; Wondergem and Bartley, 2009; Neumann and Copello, 2011; Melanaphy et al., 2016), menthol induces Ca^2+^ release from intracellular stores. In addition, menthol has been shown to induce activation of IP3 receptors (Melanaphy et al., 2016), and RyR1 ryanodine receptors with an approximate EC_50_ of 1 mM (Palade, 1987; Neumann and Copello, 2011). Thus, it is likely that some of the menthol actions are due to enhancement of Ca^2+^-induced inactivation of VGCCs and/or activations of second messenger pathways as a result of alteration in intracellular Ca^2+^ levels.

### Voltage-gated potassium channels

Voltage-gated K^+^ channels (VGKCs) are important membrane proteins embedded into lipid bilayer membrane. Stimulation of VGKCs by depolarizing membrane potentials leads to opening of these channels and causes the hyperpolarization of excitable cells. The VGKCs are broadly distributed throughout the mammalian tissues and play vital roles in dampening cellular excitability under several pathological and physiological conditions (Maljevic and Lerche, 2013; Tian et al., 2014). Some of the physiological functions of K^+^ channels include determining the action potential duration, changing the interspike intervals during repetitive firing of the heart and neurons, secreting K^+^ in epithelial tissue, and causing smooth muscle contractions (Maljevic and Lerche, 2013; Tian et al., 2014). These channels are composed of four subunits, gathered in the cell membranes as tetrameric structures. Due to the large number of different genes present, auxiliary β-subunits and metabolic regulation, there is considerable functional diversity among different subtypes (González et al., 2012; Latorre et al., 2013).

Data on the effect of menthol on K^+^ channels are rather limited. In human glioblastoma cells, Ca^2+^-activated K^+^ currents were demonstrated to be stimulated by menthol (Wondergem and Bartley, 2009). Recently, using a dye sensitive to membrane potential, menthol was reported to suppress Kv7.2/3 channel subtypes that produce the M-current in neurons with IC_50_ value of 289 μM (Vetter et al., 2013). However, further investigations are needed to assess the importance of menthol as a modulator of VGKCs in various cell types.

### Transient receptor potential channels

Transient Receptor Potential (TRP) superfamily of non-selective cation channels are encoded by more than 30 distinct genes in mammals and play important roles in sensory physiology, which include contributions to thermo- and osmosensation, vision, touching, olfaction, taste, and hearing (Nilius and Szallasi, 2014). TRP channels have tetrameric structures formed by six transmembrane domain subunits and cation-selective pores, which usually show high permeability to calcium (Latorre et al., 2009). TRP channels in mammalian cells are composed of seven families of related proteins: TRPM (melastatin), TRPA (ankyrin-like), TRPV (vanilloid), TRPC (classical or canonical), TRPN (no mechanoreceptor potential C) TRPML (mucolipin), and TRPPP (polycysteine) (Clapham and Squire, 2009; Gees et al., 2012; Nilius and Szallasi, 2014). In addition to sensory physiology, these channels play diverse functional roles ranging from modification of growth cone morphology to intracellular Ca^2+^ homeostasis (Freichel and Flockerzi, 2007; Julius, 2013; Nilius and Appendino, 2013; Billeter et al., 2014). Importantly, majority of TRP channels function as polymodal sensors. These channels are activated by physical stimuli such as stretch, osmotic pressure, membrane voltage, and temperature as well as chemical stimuli, fatty acids and other membrane lipids (Bradshaw et al., 2013). Besides direct activation by physical stimuli, unliganded TRP channels can also be stimulated by G-protein coupled receptors (Veldhuis et al., 2015) and receptor tyrosine kinases (Nilius and Szallasi, 2014).

Menthol, in the concentrations ranging from 10 μM to 1 mM, has been reported to stimulate TRPM8 receptors (McKemy et al., 2002; Peier et al., 2002). Intracellular Ca^2+^ levels have been shown to be elevated by menthol in a concentration-dependent manner in HEK293 and CHO cells overexpressing TRPM8. Patch-clamp experiments in sensory neurons have demonstrated that activation of TRPM8 receptors induces outwardly rectifying, Ca^2+^ permeable cation currents that show strong resemblances to the endogenous menthol- and cold-activated currents (McKemy et al., 2002; Peier et al., 2002). An examination of the voltage dependence of currents mediated by TRPM8 receptors showed that cold and menthol employ the similar mechanism to activate this channel. It appears that both menthol and cold shift the voltage-dependent activation curve of TRPM8 to more physiological potentials (Voets et al., 2004).

Structures of TRP channels are tetrameric and composed of four subunits containing six transmembrane segments (TM1–TM6). Mutation of residues sensitive to potential changes in the TM4 and the TM4-TM5 linker effects menthol- and cold-sensitivity of TRPM8 receptors (Voets et al., 2007). Random mutagenesis screening studies indicated that tyrosine 745, located in the middle of putative transmembrane segment 2, is as a crucial amino acid residue for the menthol sensitivity of mouse TRPM8 receptor (Bandell et al., 2006). Receptor-channel complex that contains TRPM8-Y745H mutation was found to be not sensitive to menthol, but retained the voltage and cold sensitivity of the wild-type channel. In another study, it was proposed that single TRPM8 channel can independently bind up to four menthol molecules, and that bound menthol molecules cause a similar energetic stabilization of the open channel (Janssens and Voets, 2011). Menthol sensitivity of TRPM8 receptors has also been shown to be influenced by post-translational modifications such as glycosylation (Pertusa et al., 2012). It seems that reduction of TRPM8 glycosylation causes a significant decrease in the menthol responsiveness of TRPM8 receptor (Pertusa et al., 2012). Another feature that appears to influence sensitivity of TRPM8 receptors to menthol is the bilayer lipid structure of the plasma membranes. Interestingly, disturbance by methyl-β-cyclodextrin of lipid rafts has been demonstrated to increase stimulation of TRPM8 receptors by menthol (Morenilla-Palao et al., 2009).

Notably, the interaction between menthol and TRPM8 receptor is not limited to excitable cells. Various non-excitable cancer cells expressing TRPM8 receptors has also been shown to be affected by menthol. For example, menthol has been shown to inhibit growth of human melanoma cells via activation of TRPM8 receptors (Slominski, 2008; Yamamura et al., 2008). Similarly, menthol has been reported to induce cell death via the TRPM8 receptor in the human bladder cancer cell line T24 (Li et al., 2009) indicating that menthol modulation of TRPM8 receptors may have some clinical implications as well.

Besides menthol, in the concentrations ranging from 100 μM to 10 mM, many monoterpenes with structures similar to menthol, including carvone, isopulegol, and euganol (Bandell et al., 2004), linalool, geraniol, eucalyptol, citronellal (Behrendt et al., 2004), and menthone (McKemy et al., 2002) also stimulate TRPM8 receptors (for a review Oz et al., 2015). However, actions of these compounds, compared to menthol, are less described and it is not clear if they utilize the same binding site as menthol on the TRPM8 receptor.

Among naturally occurring TRP ligands some extent of promiscuity exists. For example, the monoterpene menthol, generally considered a TRPM8-specific agonist, was reported to activate TRPV3 (Macpherson et al., 2006; Vogt-Eisele et al., 2007) and TRPA1 (Karashima et al., 2007) receptors. It appears that menthol in the range of submicromolar to low-micromolar concentrations, causes activation of TRPA1 channel, whereas at higher concentrations menthol leads to a reversible blockade of the channel (Karashima et al., 2007). Menthol has also been shown to induce membrane depolarizations by activating TRPA1 channels in interstitial cells of Cajal (Kim et al., 2016). While activation of TRPM8 by menthol occurs with an EC_50_ of approximately in the range of 4–80 μM (McKemy et al., 2002; Peier et al., 2002; Behrendt et al., 2004), higher menthol concentrations are required for modulation of other thermosensitive TRP channels.

Earlier studies have also shown TRP channel-independent actions of menthol. In various cell types, including skeletal muscle sarcoplasmic reticulum (Palade, 1987; Neumann and Copello, 2011; EC_50_ = 0.9–1 mM), tracheal epithelial cells (Takeuchi et al., 1994; 100 μM–1 mM) human leukemia cells (Lu et al., 2006; 25 to 100 μM), gliablastoma cells (Wondergem and Bartley, 2009; 100 μM), commonly used cell lines (HEK-293, LNCaP, CHO, and COS, Mahieu et al., 2007; 100 μM–1 mM) and dorsal horn neurons (Tsuzuki et al., 2004, 100 μM–1 mM), menthol has been shown to increase intracellular Ca^2+^ levels independently of TRP-channel activation.

Menthol also directly inhibits the activity of several enzymes (Kim et al., 2008, 2009). In HEK-293 and HeLa cells, menthol, in the concentrations of 0.3–1 mM, causes significant inhibition of P2Y purinoceptor-mediated or histamine receptor-mediated cytosolic Ca^2+^ mobilization (Kim et al., 2008). The results of further biochemical experiments in this study indicated that menthol inhibits the activity of PLC directly. Furthermore, the authors have shown that ADP-stimulated aggregation of human erythrocytes was blocked by menthol, suggesting that menthol can be a clinically useful agent to prevent platelet aggregation. Similarly, in PC-3 cells, phosphorylation of c-jun N-terminal kinase (JNK) was significantly potentiated by menthol (Kim et al., 2009).

In human airway Calu-3 epithelial cells that do not express TRPM8 channels, menthol (0.1–1 mM) heterologously regulates anion transport through cAMP-independent mechanisms by potentiating the cystic fibrosis transmembrane conductance regulator (CFTR)- mediated Cl^−^ transport (EC_50_ = 190 μM) and by down regulating Na^+^-K^+^-2C1^−^ transporter activity (Morise et al., 2010). It is concluded that the actions of menthol on actin cytoskeleton which interacts with these anion transporters mediates the observed effects of menthol on epithelial Cl- transport and suggested that menthol may have beneficial actions in treatment of cystic fibrosis (Morise et al., 2010). Similarly, menthol has been shown to interfere with tubulin depolymerization and promote apoptosis (Faridi et al., 2011). Overall, these studies indicate that menthol causes several effects on intracellular Ca^2+^ levels and other cellular events independent of TRP-channels.

### GABA and glycine receptors

Receptors for inhibitory neurotransmitters γ-amino butyric acid (GABA) and glycine (Gly) belong to pentameric ligand-gated ion channel family. These receptors respond to GABA and Gly by opening a chloride-selective central pore. In the central nervous system, GABA is the major inhibitory neurotransmitter. It is synthesized in GABAergic neurons and in response to action potential, it is released from presynaptic buttons into the synaptic cleft. Subsequent to its release, GABA acts primarily at two different types of receptors: first the ionotropic GABA_A_ receptors and secondly, the metabotropic, G-protein coupled GABA_B_ receptors. In case of GABA_A_ receptors, binding of GABA to its receptor causes a conformational change in its structure and leads to the opening of the channel. Activation of GABA_A_ receptors causes hyperpolarization and eventually the suppression of neuronal activity (Fritschy et al., 2012; Sigel and Steinmann, 2012). On the other hand, the receptors for Gly are located primarily in the spinal cord and brain stem. Following its release from nerve endings, Gly binds to post-synaptic Gly receptor and opens a chloride channel intrinsic to the receptor (Yevenes and Zeilhofer, 2011; Dutertre et al., 2012).

In recent years, the actions of menthol and structurally related monoterpene analogs on GABA_A_-receptors have attracted considerable attention. Notably, in majority of earlier investigations menthol was found to upregulate the function of GABA_A_-receptors. In a previous investigation, actions of menthol and its structural analogs (monoterpenoid alcohols and ketones) were examined on recombinant human GABA_A_ (α1β2γ2s) and glycine (α1 homomers) receptors expressed in *Xenopus* oocytes (Hall et al., 2004). Monoterpences, in the concentration range of 10–300 μM, caused a significant increase of GABA-induced currents in the following order: (+)-menthol > (−)-menthol > borneol > menthone = camphor = carvone. In this study menthol was found to be activated stereoselectively (Hall et al., 2004). Importantly, menthol up to concentration of 1 mM did not induce any ion current indicating that this compound does not have direct agonist activity on GABA_A_ receptor. Given its marked effects (e.g., at 100 μM, GABA and glycine EC_20_ responses increased by 496 and 135%, respectively) potentiating effects of menthol was further investigated. Menthol, 100 μM, reduced EC_50_ values for Gly and GABA from 98 to 75 μM, and from 82 to 25 μM respectively, without a significant effect on maximal responses (Hall et al., 2004).

In further studies on human α1β2γ2s GABA_A_ receptors expressed in *Xenopus laevis* oocytes (Watt et al., 2008), it was reported that when these compounds were co-applied with sub-maximal (EC_20_) GABA concentrations, the amplitudes of current responses were increased in concentration-dependent manner in the following order: (+)-menthol > isopulegol > isomenthol > α-terpineol >> cyclohexanol. Importantly, flumazenil (a benzodiazepine antagonist) did not reverse menthol enhancement of GABA-induced currents while GABA responses activated by propofol (50 μM) were significantly suppressed by menthol (50 μM). GABA_A_ receptors containing β2 subunits with either a point mutation of a tyrosine to a tryptophan at the 444 position (TM-4) or a methionine residue to a tryptophan at the 286 position (in transmembrane domain 3, TM-3) are found to be insensitive to propofol modulation. Potentiation of GABA EC_20_ currents by menthol were equally eliminated in GABA_A_ α1β2(M286W)γ2s and α1β2(Y444W)γ2s receptors while potentiation by barbiturates, benzodiazepines, and steroids remained unaltered (Watt et al., 2008). The findings of this study showed that menthol acts on GABA_A_ receptors via a site related to propofol modulation but distinct from action sites for barbiturates, benzodiazepines, and steroids. Finally, application of menthol, in an *in vivo* tadpole assay, resulted in a loss of righting reflex with an EC_50_ of 23 μM (~10-fold less potent anesthesia than propofol). Thus, it is possible that menthol shares general anesthetic action with propofol (Watt et al., 2008) possibly by acting at similar binding sites on the GABA_A_ receptor.

In radioligand binding studies, specific binding of [^3^H]-flunitrazepam to GABA_A_ receptors in primary cultures of mouse cortical neurons was not altered by up to 500 μM concentrations of menthol (García et al., 2006). In another investigation, it was reported that only (+)-menthol, among the five menthol stereoisomers studied, was found to be active in potentiating the binding of [^3^H]-flunitrazepam, an allosteric ligand for GABA_A_ receptor (Corvalán et al., 2009) while (+) and (−)-neomenthol were found to be inactive. In another radioligand binding study, monoterpenes such as carvacrol, citronellic acid, 1,8-Cineole, thymol, and pulegone significantly increased the specific binding of [^3^H]-tbutylbicycloorthobenzoate (TBOB), a non-competitive inhibitor of picrotoxin. On the other hand, menthol and other monoterpenoids such as vanillin, camphor, and safrole, significantly suppressed the binding of [^3^H]-TBOB in housefly head membrane preparations (Tong and Coats, 2012).

In another investigation, it was shown that the excitability of cultured rat hippocampal neurons were inhibited menthol (Zhang et al., 2008). In addition, menthol significantly inhibited the epileptic activity induced by pentylenetetrazole injection and electrical kindling in *in vivo* models (Zhang et al., 2008). It was reported that menthol not only increased the currents activated by low concentrations of GABA but also *directly* induced currents mediated by GABA_A_ receptors in cultured hippocampal neurons. In addition, menthol significantly increased tonic GABAergic inhibition in the CA1 region of rat hippocampal slices. But phasic GABAergic inhibition remained unaffected. The structure-effect relationship of menthol suggested that hydroxyl group plays an important role in the enhancement of tonic GABA_A_ receptors by menthol (Zhang et al., 2008). Another study examined the actions of menthol on respiratory rhythm generation in the brainstem-spinal cord preparations from newborn rats (Tani et al., 2010). It was reported that menthol, by directly activating tonic GABA receptors, caused a significant inhibition of burst generation in pre-inspiratory neurons. In a recent study (Lau et al., 2014), the effects of menthol on GABA_A_ receptors were examined in periaqueductal gray (PAG) neurons of midbrain slices It was found that menthol (150–750 μM) induced prolongation of spontaneous GABA_A_ receptor-mediated inhibitory post-synaptic currents in a concentration-dependent manner, but non-NMDA receptor-mediated excitatory post-synaptic currents remained unaltered. Effects of menthol were not changed by antagonists of TRPM8 and TRPA1 receptors, flumezanil, a benzodiazepine antagonist, and tetrodotoxin, sodium channel blocker. A tonic current, which was sensitive to the bicuculline and picrotoxin (both are GABA_A_ receptor antagonists) was also enhanced by menthol. These results indicated that both synaptic and extrasynaptic populations of GABA_A_ receptors are positively modulated by menthol in native PAG neurons.

The action of menthol on the Gly receptors are relatively less studied in comparison to GABA_A_ receptors. In an earlier investigation (Hall et al., 2004), actions of menthol were studied on homomeric α1 glycine receptors expressed in *Xenopus* oocytes. It was reported that the function of Gly receptors was significantly enhanced by both (+) and (−) enantiomers of menthol and borneol.

### Serotonin type-3 receptors

Serotonin type-3 (5-HT_3_) receptors belong to Cys-loop family of ligand-gated ion channel family and therefore differ structurally and functionally from other G-protein coupled serotonin receptors. Human 5-HT_3_ receptors are proposed to play important roles in neurodevelopment, nociception, psychiatric disorders such as depression, and motility of gastrointestinal system (Lummis, 2012; Engel et al., 2013). The actions of menthol on the functional properties of 5-HT_3_ receptors were studied in recent investigations (Heimes et al., 2011; Ashoor et al., 2013b; Walstab et al., 2014; Ziemba et al., 2015). Actions of menthol on 5-HT_3_ receptors were studied by Heimes et al. (2011) utilizing three different *in vitro* models: isotonic contractions of the isolated rat ileum and equilibrium competition binding studies using [^3^H]GR65630, a 5-HT_3_ receptor antagonist, and [^14^C] guanidinium influx into N1E-115 cells which express 5-HT_3_ receptors. Application of menthol suppressed [^14^C]guanidinium influx through 5-HT_3_ receptors as well as 5-HT induced contractions of the ileum. However, specific binding of [^3^H]GR65630 to 5-HT_3_ receptor was not altered by menthol. In this investigation, it was suggested that antiemetic actions of menthol occur at least partly by acting as a negative allosteric modulator of the 5-HT_3_ receptors; in other words, by binding to an allosteric modulatory site which is distinct from the binding site of serotonin. In another recent investigation, Ashoor et al. (2013b) studied the actions of menthol on the functional properties of human 5-HT_3A_ receptors expressed in *X. laevis* oocytes. Menthol reversibly suppressed 5-HT-activated inward currents in a concentration-dependent manner with an IC_50_ value of 163 μM. The time course of the inhibitory effect of menthol was slow and reached a steady-state level within 10–15 min. However, the effect did not involve G-proteins, since GTPγS activity remained unchanged. In addition, pretreatment with pertussis toxin, which inhibits G_i_ and G_o_ proteins, did not alter the extent of menthol inhibition. Racemic, (−), (+) menthols inhibited 5-HT_3_ currents to the same extent suggesting that the effects of menthol on 5-HT_3_ receptors are not stereoselective. Increasing concentrations of 5-HT did not reverse the inhibition induced by menthol. Moreover, menthol did not affect specific binding of the [^3^H]GR65630, indicating that this compound acts as a non-competitive antagonist of the 5-HT_3_ receptor. Finally, in acutely dissociated nodose ganglion neurons, 5-HT_3_ receptor-mediated currents were inhibited by menthol. The results of this study indicated that in both heterologous expression systems and in neurons, menthol acts as a negative allosteric modulator of 5-HT_3_ receptors. In another recent study actions of menthol was investigated in HEK293 cells expressing human recombinant homomeric 5-HT_3A_- and heteromeric 5-HT_3AB_ receptors using a luminescence-based Ca^2+^ assay, membrane potential assay, and radioligand binding assay (Walstab et al., 2014). The results of this study indicated that (−) isomer of menthol inhibited 5-HT_3_ receptors with an IC_50_ of 20 μM in a non-competitive manner. Importantly, the potency of (+)-menthol was significantly less than that of the (−) stereoisomer. In addition, (+)-menthol, compared to (−) menthol, was significantly less potent on 5-HT_3A_ vs 5-HT_3AB_ receptors. Overall, (−)-menthol was 11-fold more potent toward the homomeric 5-HT_3A_ receptor. Above-mentioned studies demonstrate that menthol is a negative allosteric modulator of 5-HT_3_ receptors. Similar to menthol, thujone, another monoterpene chemically related to menthol, has also been demonstrated to suppress the function of 5-HT_3_ receptors (Deiml et al., 2004).

### Nicotinic acetylcholine receptors

The nicotinic acetylcholine receptors (nAChRs) are cation-permeable ion channel-receptor complex activated by the neurotransmitter acetylcholine. The nAChRs is a member of Cys-loop receptor family, which also includes the GABA_A_, GABA_C_, Gly, and serotonin 5-HT_3_, receptors. The nAChRs also play important roles in several physiological functions and pathological conditions, including the modulation of neurotransmitter release, the secretion of hormones, and regulation of neuronal excitability (Dani and Bertrand, 2007; Dineley et al., 2015). The nAChRs are expressed throughout the neurons of central and peripheral nervous systems, as well as cells of the other peripheral tissues (Dani and Bertrand, 2007; Albuquerque et al., 2009). Currently, nine different nAChR subunits have shown to be expressed (α2-7 and β2-4) in the mammalian brain. The subunits combinations, as either homomeric or heteromeric complexes, can occur resulting in functionally diverse pentameric receptors (Albuquerque et al., 2009; Dineley et al., 2015). The main receptor subtypes expressed in the brain are α7 containing subunits and those composed of both α and β subunits, including the α4β2^*^ and α3β4^*^ subtypes; the ^*^ designates that these receptors can contain other α and β subunits as well. In searching for new selective molecules that modulate the functional properties of nACh receptors, naturally produced chemicals have shown to be a good source at least for generations of structural models (Daly, 2005; Romanelli et al., 2007).

In tobacco industry, menthol is commonly employed to mask the unpleasant tobacco test, increase the ease of smoking and offer a cooling sensation that appeal to many smokers (Ahijevych and Garrett, 2004). Moreover, it has been reported that menthol is present in 90 percent of tobacco products in varying concentrations (Foulds et al., 2010). Menthol, as an additive to tobacco products, has come under scrutiny following several FDA reports (Benowitz and Samet, 2011), indicating that menthol can enhance smoking behavior and stimulate adverse effect of smoking on health (Kabbani, 2013; Wickham, 2015). The results of epidemiological studies suggest that smoking of mentholated cigarettes is more widespread in ethnic and racial minority populations (Ahijevych and Garrett, 2010; Foulds et al., 2010). It is also important to note that an association between a difficulty in quitting smoking and smoking mentholated cigarettes is also greater in ethnic and racial minority populations as well as young smokers (Foulds et al., 2010). Therefore, it is essential to investigate the cellular and molecular mechanisms of interaction between nAChRs and menthol.

Functional interaction between nicotinic receptors and menthol has been reported earlier both *in vitro* and *in vivo* investigations (Dessirier et al., 2001; Ruskin et al., 2008; Willis et al., 2011; Hans et al., 2012; Ashoor et al., 2013a; Amato et al., 2014b). In an earlier study, it was found that menthol significantly reduces sensory perception and irritation caused by nicotine (Dessirier et al., 2001) and by inhalation of cigarette smoke (Willis et al., 2011). In addition to these findings, nicotine-induced decreases in body temperature, as a result of cutaneous vasodilation, are reduced considerably following the administration of both acute and chronic menthol (Ruskin et al., 2008). In a recent investigation, the extent of gastric relaxation induced by menthol was considerably decreased in the presence of hexamethonium, a nAChR blocker (Amato et al., 2014b). Menthol, at concentrations that did not alter gastric tone, decreased the contraction caused by nAChR agonist, dimethylphenylpiperazinium. The co-application of hexamethonium and phentholamine, α-adrenergic receptor antagonist or hexamethonium and atropine, muscarinic receptor antagonist, did not cause any additive decrease of the menthol-induced relaxation. In this study, authors concluded that the interaction between nAChR and menthol is likely to be an important stage for menthol-induced relaxation of gastric muscle (Amato et al., 2014b).

Electrophysiological studies investigating the effects of menthol on different nAChR subtypes have also been conducted (Hans et al., 2012; Ashoor et al., 2013a). It was reported that, in trigeminal neurons, nicotine-induced whole-cell currents through nACh receptors was reversibly inhibited with an IC_50_ of 111 μM. Single channel experiments on human α4β2 nAChR expressed in HEKtsA210 cells indicated that menthol induced a decrease of channel open time, increase in single channel amplitude, and increase of channel closed time leading to overall reduction in single channel currents. Moreover, menthol did not alter the potency (nicotine's EC_50_ value) on recombinant human α4β2 nAChRs but induced a substantial decrease in the efficacy of nicotine. All together, these results demonstrated that menthol is a negative allosteric modulator of α4β2 nAChRs (Hans et al., 2012). Clinical studies employing positron emission tomography scanning with the α4β2 radioligand indicated that, in line with the results of *in vitro* studies, menthol smokers have 9–28% higher α4β2 nAChR densities than non-menthol smokers across regions (Brody et al., 2013).

In a recent study, the effect of long-term menthol application was investigated on midbrain neurons containing nAChRs (Henderson et al., 2016). Menthol alone increased the number of α4 and α6 nAChR subunits in midbrain dopaminergic neurons from mice expressing fluorescent nAChR subunits. However, this upregulation did not occur in midbrain GABAergic neurons suggesting that chronic menthol application produced a cell-type-selective upregulation of α4^*^ nAChRs. These findings complemented that of chronic nicotine alone, which upregulates α4 subunit-containing (α4^*^) nAChRs in GABAergic but not dopaminergic neurons. Further studies in cultured midbrain neurons and mouse brain slices indicated that menthol decreased dopaminergic neuron firing frequency and altered dopaminergic neuron excitability following nAChR activation. Furthermore, exposure to menthol before nicotine stopped nicotine reward-related behavior in mice. In neuroblastoma cells transfected with fluorescent nAChR subunits, exposure to menthol (500 nM) alone also increased the number of nAChRs and favored the formation of (α4)3(β2)2 nAChRs. This effect contrasted with the action of nicotine, which favors (α4)2(β2)3 nAChRs (Henderson et al., 2016). Menthol alone also increases the number of α6β2 receptors that exclude the β3 subunit. Thus, it appears that lower-sensitivity α4^*^ and α6 subunit-containing nAChRs were stabilized by menthol. The suppression of nicotine reward-related behavior may be mediated through menthol's ability to stabilize lower-sensitivity nAChRs and alter dopaminergic neuron excitability. Overall these studies indicated that menthol increases the number of nAChRs in the mouse brain at a dose that matches nicotine in its ability to increase nAChR number. Menthol also changes the function of midbrain dopamine neurons, and prevents behaviors related to nicotine reward. These findings suggest that menthol is more than an “inert” additive to tobacco and it is able to alter the functional properties of midbrain dopamine neurons in the mesolimbic reward pathway. In fact, another recent study indicate that menthol enhances nicotine-induced changes in nAChRs expressed on midbrain dopaminergic neurons (Henderson et al., 2017). Menthol plus nicotine upregulates nAChR number and function on midbrain dopaminergic neurons more than nicotine alone. Menthol also enhances nicotine-induced changes in dopaminergic neuron excitability. Furthermore, in a conditioned place preference assay, menthol plus nicotine produces greater reward-related behavior than nicotine alone (Henderson et al., 2017).

In addition to α4β2 containing receptors, function of α7 nAChRs was also reported to be altered by menthol. Utilizing a two-electrode voltage-clamp technique, it was demonstrated that menthol reversibly inhibited human α7-nACh receptors expressed in *Xenopus* oocytes (Ashoor et al., 2013a). Inhibitory effect of menthol did not involve the activation of endogenously expressed Ca^2+^-dependent Cl^−^ channels in oocytes and the effect was not dependent on changes in the membrane potential. Moreover, increasing the concentrations of ACh did not alter the inhibition by menthol. In addition, the specific binding of α-bungarotoxin was not changed by menthol. Furthermore, studies of α7-nACh receptors endogenously expressed in neuronal cells indicated that menthol suppresses Ca^2+^ transients mediated by the activation of α7-nACh receptors in the cell body and neurite. Over all, these findings indicate that menthol non-competitively inhibits the function of α7-nACh receptors (Ashoor et al., 2013a).

In addition to centrally located α7 and α4β2 nAChRs, the effects of menthol have also been reported on α3β4, the major nicotinic subtype expressed in sensory nerves (Ton et al., 2015). In this study, menthol markedly suppressed the activity of nAChR as assessed by ^86^Rb^+^ efflux, Ca^2+^ imaging, and voltage-clamp experiments. Menthol inhibited the function of nAChRs in a voltage-independent manner. In addition, menthol decreased the mean open time of single channels without altering their conductance, disagreeing with a simple channel-blocking action. Furthermore, the recovery of nAChRs from desensitization menthol was significantly slowed or prevented by menthol, suggesting that menthol probably stabilizes a desensitized state of nAChRs. It was also demonstrated that menthol at concentrations up to 1 mM did not interact with the orthosteric nAChR binding site labeled by [^3^H] epibatidine, indicating that menthol causes desensitization of α3β4 nAChRs by an allosteric mechanism (Ton et al., 2015).

In conclusion, in both neural and non-neural nACh receptors, menthol presented a significant activity on the function of nACh receptor that requires further investigations. Drug development efforts have recently focused on direct manipulation of α7 and α4β2 nACh receptors. Menthol and related monoterpenes could be helpful in developing compounds that influence different nAChR subtypes or provide high selectivity for these subtypes.

## Relevant menthol concentrations

In the literature, there are some pharmacokinetic studies available for menthol. In an earlier study, the no-observed-adverse-effect-level after oral intake was found to be 667 mg/kg/day (National Toxicology Program, 1979). The lethal menthol concentration in orally fed mice was reported to be in the range of 2,900–6,000 mg/kg body weight, indicating that menthol, at the doses used in most *in vitro* studies (10 μM to 10 mM) is well-tolerated. In another study, the plasma menthol concentration rose to 20 μM within 1 h in rats that have been administered 400 mg of menthol/kg body weight I.P. (Spichiger et al., 2004). In an earlier study in rodents, it was reported that the average nasal tissue concentration of inhaled menthol applications (at 0.65 μM in vapor or 16 ppm) was about 150 μM (Willis et al., 2011). In clinical studies, topical menthol application (30%; 1.9 M) has been employed consistently, without initiating any skin irritation or other side effects (Hatem et al., 2006). These results indicate that the effects of menthol used in the concentration range of 10 μM to 10 mM in most *in vitro* studies are likely to be pharmacologically relevant. In addition, the concentrations of menthol utilized in *in vitro* studies are below or within the range of FDA regulated concentrations utilized in creams (2% v/v; 128 mM) and over-the-counter pain rubs (4% v/v; 256 mM) (OECD, 2003).

## Mechanisms of menthol actions

Menthol, as mentioned earlier, is a highly hydrophobic compound with Log p value of 3.4 (Turina et al., 2006; Zunino et al., 2011). In line with these findings, menthol and other structurally related monoterpenes partition into biological membranes and cause significant alterations in numerous physico-chemical characteristics of lipid bilayers (Sánchez et al., 2004; Turina et al., 2006; Zunino et al., 2011). A central question has been if the effects of these hydrophobic compounds are mediated by their direct interaction with integral membrane proteins (for example ion channels and transporters) and/or by altering the physico-chemical characteristics of the lipid membranes and indirectly affecting channel function (Lee, 2011). This dichotomy demonstrates a main challenge in understanding the mechanisms of not only menthol actions but also other hydrophobic compounds such as general anesthetics (Howard et al., 2014) alcohols (for a review Howard et al., 2014; Trudell et al., 2014), terpenes (Oz et al., 2015), steroids (Hill et al., 2015), and endocannabinoids (Oz, 2006). It appears that these hydrophobic molecules bind to sites that are distinct from the sites for binding probes (radioligands or toxins) and cause significant alterations on functions of these channels (Anishkin et al., 2014; Poveda et al., 2014). It is likely that the lipophilic molecules such as menthol partition into lipid bilayer membranes and alter the gating properties of both voltage- and ligand-gated ion channels. In most *in vitro* studies, menthol and other hydrophobic monoterpenes have been shown to act on ion channels at a concentration range of 10 μM to 10 mM. In fact, this is not a unique pharmacological property of monoterpenes. Several other hydrophobic molecules, such as alcohols and general anesthetics also act at similarly high concentrations (high μM to low mM).

In earlier studies, it has been shown that all voltage-gated ion channels (VGICs) comprises a common domain of six helical transmembrane segments (S1–S6). Among these segments, S4 has been shown to have a symmetrical arrangement of charged amino acids, with each third amino acid being lysine or arginine. Hence, S4 has been thought as the key contender for the voltage-sensing segment of VGICs. Previous crystallographic examinations of KcsA K^+^ channels have shown that the gating domain of the channel is located at the lipid-protein interface (Valiyaveetil et al., 2002; Ruta and MacKinnon, 2004). It is possible that gating domains of other VGICs also have similar orientations (Lee et al., 2005; Moreau et al., 2014; Poveda et al., 2014). In this situation, hydrophobic allosteric modulators such as menthol and other monoterpenes can operate as gating modifiers by altering the energy constraints for the movement of lipid-embedded gating domains of VGICs. As a result, changes in energy requirements of gating related conformational alterations of membrane proteins can be due to menthol-induced modifications in physicochemical characteristics of membranes. In a number of previous investigations, physiochemical characteristics of lipid bilayer membranes such as membrane fluidity and membrane thickness have been shown to be modified by menthol and other related monoterpenes (Sánchez et al., 2004; Turina et al., 2006; Reiner et al., 2009; Zunino et al., 2011).

It is known that, similar to VGIC, the energetic requirements for gating-related conformational alterations of ligand-gated ion channels (LGICs) also can be changed by actions of menthol on lipid membranes. In previous investigations, it was demonstrated that even slight changes in adjacent lipid structure can cause important alterations in the function of LGICs by changing the energetics of conformational transitions in the protein structure (Fantini and Barrantes, 2009; Barrantes et al., 2010). Besides their interaction with membranes, menthol and other monoterpenes can bind directly to residues of the transmembrane domains and alter the functional properties of LGICs. For instance, a recent investigation has identified transmembrane residues that eliminate the agonist action of thymol and carvacrol on human 5-HT_3_ receptors, or confer this property on mouse 5-HT_3_ receptors that are previously insensitive to these compounds (Lansdell et al., 2015). Moreover, at least in some investigations, stereoselectivity for the effects of monoterpenes was demonstrated (Hall et al., 2004; Watt et al., 2008; Corvalán et al., 2009; Gonçalves et al., 2010; Heimes et al., 2011; Kawasaki et al., 2013; Walstab et al., 2014). Furthermore, specific amino acid residues mediating the actions of monoterpenes were identified on 5-HT_3_ (Lansdell et al., 2015) and GABA_A_ receptors (Watt et al., 2008). Thus, it is possible that both direct binding of hydrophobic compounds to ion channel residues and changing the physicochemical properties of biological membranes can contribute to overall actions of menthol and other monoterpenes on the functional properties of ion channels.

## Author contributions

MO and LA: Idea, design, writing, and submission. EE, KY, and FH: Substantial contribution to the conception, formulation, and critical revision of the manuscript. All authors gave approval for the final submission of the review and agreed be accountable for all aspects of the work.

### Conflict of interest statement

The authors declare that the research was conducted in the absence of any commercial or financial relationships that could be construed as a potential conflict of interest.

## Abbreviations

ACh: Acetylcholine
AP: Action potential
CAP: Compound action potential
CNS: Central nervous system
DRG: Dorsal root ganglion
GABA: Gamma-aminobutyric acid
GPCR: G-protein coupled receptor
VGIC: Voltage-gated ion channel
LGIC: Ligand-gated ion channel
TRP: Transient-receptor potential
TTX: Tetrodotoxin
VGCC: Voltage-gated calcium channel
VGSC: Voltage-gated sodium channel.
